# Contractualist Moral Cognition: From the Normative to the Descriptive at Three Levels of Analysis

**DOI:** 10.1002/wcs.70011

**Published:** 2025-07-10

**Authors:** Arthur Le Pargneux

**Affiliations:** ^1^ Department of Psychology Harvard University Cambridge Massachusetts USA; ^2^ Behavioural Science Group, Warwick Business School University of Warwick Coventry UK

**Keywords:** contractualism, cooperation, ethics, judgment and decision making, moral cognition

## Abstract

Contractualist moral theories view morality as a matter of mutually beneficial agreements among rational agents. Compared to its rivals in moral philosophy–consequentialism, deontology, and virtue ethics–contractualism has only recently started to attract attention in empirical work on the cognitive science of morality. Is it fruitful to adopt a contractualist lens to better understand how moral cognition works? After introducing the main contractualist theories in contemporary moral philosophy, I present five reasons to take inspiration from this family of normative theories to develop descriptive accounts of morality. Then, I review how the contractualist framework has been used to contribute to our understanding of moral cognition at three interrelated levels of analysis: Morality's evolutionary logic, its cognitive organization, and the specific cognitive processes and forms of reasoning involved in moral judgment and decision making. First, several evolutionary accounts of morality argue that its evolutionary logic must be understood in contractualist terms. Second, resource‐rational contractualism proposes that the subcomponents of moral cognition–including well‐studied rule‐ and outcome‐based mechanisms, and much less studied agreement‐based processes–are organized to efficiently approximate the outcome of explicit negotiation under resource constraints. Third, recent empirical developments suggest that three characteristically contractualist forms of reasoning–virtual bargaining, we‐reasoning, and universalization–can be involved in producing moral judgments and decisions in a variety of contexts. Beyond the traditional distinction between rules and consequences, these various research programs open a third way for the cognitive science of morality, one based on agreement.

This article is categorized under:

Psychology > Reasoning and Decision Making
Economics > Interactive Decision‐Making
Philosophy > Value

## Graphical abstract

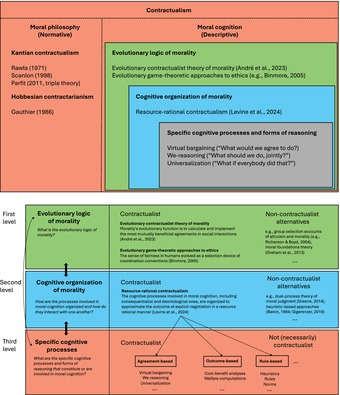

## 1 Introduction

In recent decades, the study of moral cognition has been largely shaped by the historical distinction in moral philosophy between deontology and consequentialism (Malle 2021). For deontology, morality is about rules, duties, and rights, which constitute constraints on actions (Bartels et al. 2015). For consequentialism, what is moral is what produces the best consequences, which is often understood as what impartially maximizes aggregate welfare (Everett and Kahane 2020). A third approach, which belongs to the social‐contract tradition, contractualism, views morality as a matter of mutually beneficial agreements among rational agents (Baumard 2016). Until recently, contractualism had received much less attention than consequentialism and deontology in scientific research on morality. Is it fruitful to adopt a contractualist lens to better understand how moral cognition works? Recent empirical and theoretical developments show that taking inspiration from contractualism offers great promise to better understand key aspects of the moral mind.

The first section of this paper presents contractualism in moral philosophy, introducing the main contemporary contractualist theories in normative ethics. This section also briefly outlines five reasons to take inspiration from contractualist moral theories to construct descriptive models of morality. The second section reviews how the contractualist framework has been used to study morality at three separate but complementary levels of analysis: Its evolutionary logic, its cognitive organization, and its specific cognitive processes. To begin with, some evolutionary game‐theoretic approaches to morality argue that its ultimate function is in an important sense contractualist (André et al. 2023; Binmore 2005). Next, resource‐rational contractualism proposes that the different components of the moral mind are organized to efficiently approximate the outcome of explicit bargaining processes under cognitive resource constraints (Levine, Chater, et al. 2024). Finally, recent empirical progress suggests that three contractualist cognitive processes–virtual bargaining, we‐reasoning, and universalization–can play a role in the production of moral judgments and behavior. Beyond the traditional divide between rules and consequences, these various research agendas offer a novel approach in the study of moral cognition, one in which agreements play a central role.

## 2 Contractualism in Moral Philosophy

This first section presents contractualism as a family of moral theories in normative ethics. It highlights two key elements that are common to the main contractualist approaches: the rationality of the contracting parties and the mutually beneficial character of the agreements they seek. It then introduces contractualism's main contemporary formulations in the works of Rawls, Gauthier, Scanlon, and Parfit. Lastly, it offers five reasons to take these normative moral theories as a basis for descriptive theorizing.

### 2.1 Contractualism

The term contractualism, used in its broad sense, refers to the family of moral theories that originates in the social‐contract tradition of Hobbes (1651/2018), Rousseau (1762/2004), and Kant (1739/2000), and views morality as based on some form of contract or mutual agreement (Ashford and Mulgan 2007). It is distinct from political theories of the social contract, which use the ideas of a hypothetical social contract and the consent of the governed to ground the legitimacy of political authority, rather than provide a foundation for ethics (Cudd and Eftekhari 2000). Such political theories will not be of primary concern for us here.

Contractualism comprises two related but distinct strands of thought: “Hobbesian contractualism” (also known as contractarianism)—associated with Hobbes and contemporary philosopher Gauthier (1986)—and “Kantian” contractualism—notably defended by Rawls (1971) and Scanlon (1998). For Hobbesian contractualism, morality consists in mutually advantageous cooperative behavior between self‐interested agents. By contrast, Kantian contractualists emphasize the importance of mutual respect and equality between the contracting agents. They posit that morality is concerned with the binding agreements that would result if we were to bargain as rational autonomous agents respecting one another's equal moral importance (Ashford and Mulgan 2007). Note that “contractualism” is sometimes used in a narrow sense to refer to Scanlon's theory only. Unless specified, the term contractualism will not be used in this narrow sense here.

While the above two approaches differ in important ways, two key features seem to be common to all main contractualist theories.

First is the idea that the moral acceptability of an action depends on appropriate consideration of the interests of all relevantly affected parties, which are taken to be *rational* or *reasonable*. As such, Rawls (1971, 19) is concerned with the principles of justice that “rational persons concerned to advance their interests would consent to as equals” while Scanlon (2013, 601) puts emphasis on the “notion of reasonable agreement”. Similarly, Gauthier (2013b, 575) refers to “[…] unanimous agreement among rational persons who were choosing the terms on which they would interact with each other. And this agreement is the basis of morality.” Thus, the contracting parties posited by contractualist theories are understood to be rational in an important sense^1^.

Second is the idea that morality is grounded in the pursuit of mutual benefit or mutual advantage. As Baumard (2016) explains it, for contractualist philosophers everything happens as if our moral duties resulted from the successful negotiation of mutually advantageous contracts that respect the interests of each of the contracting parties. And indeed, Rawls views society as “a cooperative venture for mutual advantage” (Rawls 1971, 4). Similarly, Gauthier explicitly specifies that “for a contractarian, morality requires a context of mutual benefit” (Gauthier 1986, 17) and that “a necessary condition of such agreement is that its outcome be mutually advantageous” (Gauthier 1986, 14). And while Scanlon does not explicitly refer to notions of benefit or advantage, the principles identified by his theory are expected to be mutually advantageous in an important sense. In particular, he acknowledges that, “In many cases, gains and losses in well‐being […] are clearly the most relevant factors determining whether a principle could or could not be reasonably rejected” (Scanlon 1998, 215). Overall, for contractualists, acting morally means acting in a mutually advantageous way (Baumard 2016), and contractualist agreements are taken to be mutually beneficial.

### 2.2 Main Contemporary Contractualist Theories

Having introduced contractualism, we can turn to its main formulations in contemporary ethics, as proposed and defended by Rawls, Gauthier, Scanlon, and Parfit.

#### 2.2.1 Rawls

Rawls (1971) aims to identify the principles of justice that self‐interested, free, and rational persons would accept in an initial position of equality, the “original position”. These principles of justice are chosen behind a “veil of ignorance” in which agents do not know their place in society, their class, their social status, their personal characteristics (e.g., intelligence and health), nor their level of wealth and material possessions. This means that, in the original position, the contracting parties are taken to be rational and mutually disinterested. And their choice of principles of justice is the result of a fair agreement or bargain because, behind the veil of ignorance, no one can design principles that would be advantageous only for them, which is why Rawls calls his view *justice as fairness*.

Rawls proposes that, under such conditions, the following principles would be chosen. According to the first principle, each person should be assigned the same basic set of rights, duties, and liberties, equally. The second principle posits that social and economic inequalities can only be viewed as just if there is equality of opportunity and such inequalities are to the greatest benefit of the least advantaged members of society (Wenar 2008). These two principles are obtained by applying the maximin principle to the problem of social justice, in which the alternative whose worst outcome is superior to the worst outcomes of the other options under consideration is adopted (Rawls 1971).

While *justice as fairness* is primarily a political theory, Rawls explicitly indicates that his method can be used to develop a full contract theory, including a theory of morality and ethics which he calls “rightness as fairness” (Rawls 1971, 17). This view of morality can be helpfully summarized as follows: “Everyone ought to follow the principles to whose universal acceptance it would be rational in self‐interested terms for everyone to agree, if everyone had to reach this agreement without knowing any particular facts about themselves or their circumstances” (Parfit 2011, 349). Or, more concisely: “something is right…when an ideally rational and impartial spectator would approve of it” (Parfit 2011). Relative to other contractualist accounts, a key aspect of Rawls' theory is its emphasis on impartiality.

#### 2.2.2 Gauthier

Gauthier (2013b, 575) grounds morality in individual rationality: “The foundational crisis of morality is thus resolved by exhibiting the rationality of our compliance with mutual, rationally agreed constraints on the pursuit of our desires, aims, and interests.” In the spirit of the contractarian approach started by Hobbes (1651/2018), he holds that we all have much to gain from cooperation and that it is rational to impose moral constraints upon ourselves in order to reap the benefits of mutually advantageous cooperation.

For Gauthier, (self‐interested) agents that accept such moral constraints—which he calls, “constrained maximizers”—will be better at expected utility maximization than “straightforward maximizers”—(self‐interested) agents that do not accept moral constraints. This is because, over time, straightforward maximizers will be excluded from certain mutually advantageous cooperation opportunities. He concludes that constrained maximization is rational and thus that moral constraints can be derived solely by appealing to rational self‐interest (Gauthier 1986).

Originally, the preferred bargaining mechanism proposed by Gauthier (1986) was minimax relative concession (or maximin relative benefit), in which each agent tries to minimize the maximum relative concession that they make from their ideal outcome relative to the concession that other contracting parties make. More recently, Gauthier (2013a) has also proposed an alternative view of the problem in which he instead sees rational agents as Pareto‐maximizers. Under this approach, agents want to reap all the benefits available through cooperation and they do so by trying to achieve Pareto‐optimal outcomes, situations in which no agent can increase their own payoff without making another agent worse off (Cudd and Eftekhari 2000). Compared to other contractualist accounts, Gauthier's theory gives more importance to individual rationality.

#### 2.2.3 Scanlon

For Scanlon (1998), morality is based on a hypothetical agreement between free and equal agents that mutually respect each other. His contractualism puts emphasis on justifiability, as it is concerned with explaining the justificatory status of moral properties and their motivational force by appealing to the notion of reasonable agreement (Scanlon 2013). Under this view, wrongness precisely consists in unjustifiability: it is the property of being unjustifiable (Ashford and Mulgan 2007), and people can reasonably reject a principle if they have a reasonable complaint to the principle. Good reasons for complaints are plural and may include, for example, consequences on one's well‐being, unfairness, or violation of one's freedom or autonomy (Southwood 2009).

As such, one of Scanlon's central ideas is that morality is fundamentally about principles that we can justify to others, in other words that other individuals could not reasonably reject. This leads to the following formulation of his contractualism: “An act is wrong if its performance under the circumstances would be disallowed by any set of principles for the general regulation of behaviour that no one could reasonably reject as a basis for informed, unforced, general agreement” (Scanlon 1998, 153). Parfit (2011, 360) concisely summarizes Scanlon's view as follows: “Everyone ought to follow the principles that no one could reasonably reject.” Relative to other views, Scanlon's contractualism puts special emphasis on justifiability.

#### 2.2.4 Parfit

Parfit is not a defender of contractualism per se. Instead, he argues for a “triple theory” of morality that reconciles what he takes to be the best versions of deontology, consequentialism, and contractualism. He holds that, more often than not, these three families of theories agree with each other and that deontologists, consequentialists, and contractualists are, in some sense, attempting to climb the same mountain from three different sides (Parfit 2011).

In order to derive his triple theory, he argues that Scanlonian and Kantian contractualism can be combined: “the principles that no one could reasonably reject are the same as the principles that everyone could rationally will to be universal laws” (Parfit 2011, 412). He also proposes that the best version of Kantian contractualism implies rule consequentialism: “everyone ought to follow the principles whose universal acceptance would make things go best” that is, the “optimific” principles (Parfit 2011, 375). Combining such concerns for consequences with Kantian universalizability and Scanlonian reasonable rejection leads to the following formulation of the triple theory: “An act is wrong just when such acts are disallowed by the principles that are optimific, uniquely universally willable, and not reasonably rejectable” (Parfit 2011, 25). Compared to previously presented theories, the contractualist aspects of Parfit's theory give special importance to universalizability.

#### 2.2.5 Summary

As with deontology (e.g., Kantian deontology^2^, divine command theory, deontological pluralism) and consequentialism (e.g., act utilitarianism, rule utilitarianism), contractualist theories come in a variety of forms and, within the same family of theories, specific formulations can be incompatible with one another. But whether they put more emphasis on impartiality (Rawls), individual rationality (Gauthier), justifiability (Scanlon), or universalizability (Parfit), all of them view morality as a matter of mutually beneficial agreements between rational agents, see Table 1. And it seems that we can sum up the central ideas of contractualism as follows: for contractualist theories, ethics is a matter of (hypothetical) agreements and morality is about acting according to what would be agreed by rational agents in search of mutual benefit (Le Pargneux et al. 2024).

**TABLE 1:** Overview of the main contractualist theories in contemporary moral philosophy and the main descriptive approaches in moral cognition at three interconnected levels of analysis: Evolutionary logic, cognitive organization, and specific cognitive processes.


Rawls	“Everyone ought to follow the principles to whose universal acceptance it would be rational in self‐interested terms for everyone to agree, if everyone had to reach this agreement without knowing any particular facts about themselves or their circumstances” (Parfit 2011, 349)
Gauthier	“Morality, we shall argue, can be generated as a rational constraint from the non‐moral premisses of rational choice.” (Gauthier 1986, 4) “Moral principles are introduced as the objects of fully voluntary ex ante agreement among rational persons.” (Gauthier 1986, 9)
Scanlon	“An act is wrong if its performance under the circumstances would be disallowed by any set of principles for the general regulation of behaviour that no one could reasonably reject as a basis for informed, unforced, general agreement” (Scanlon 1998, 153)
Parfit	“An act is wrong just when such acts are disallowed by the principles that are optimific, uniquely universally willable, and not reasonably rejectable” (Parfit 2011, 25)
**Contractualism in moral cognition**	
*Evolutionary logic of morality*	
Evolutionary contractualist theory of morality	“Moral cognition computes reciprocal obligations that would maximize mutual benefit if each partners complied to them, and tags as morally wrong the behaviors that violate these reciprocal contracts.” (André et al. 2023, 1) “[…] the moral sense has been shaped by natural selection to find every conceivable way for a set of individuals to generate mutual benefits” (André et al. 2023, 9)
Binmore	“[…] fairness norms then become explicable as an equilibrium selection device that selects one of the many efficient equilibria of a society's game of life. It is suggested that the deep structure of such fairness norms is captured by John Rawls' notion of the original position, and is therefore universal in the human species. On the other hand, the standard of interpersonal comparison needed as an input to the original position is culturally determined.” (Binmore 2005, 9)
*Cognitive organization of morality*	
Resource‐rational contractualism	“From a contractualist perspective, ideal moral judgments are those that would be agreed to by rational bargaining agents […] As a practical matter, however, investing time and effort in negotiating every interpersonal interaction is unfeasible. Instead, we propose, people use abstractions and heuristics to efficiently identify mutually beneficial arrangements. We argue that many well‐studied elements of our moral minds […] can be naturally understood as resource‐rational approximations of a contractualist ideal.” (Levine, Chater, et al. 2024, 1)
*Specific cognitive processes and forms of reasoning*	
Virtual bargaining	“What would we agree to do, if we could negotiate?”
We‐reasoning/joint commitments	“What should *we* do, jointly?”
Universalization	“What if everybody did that?”

### 2.3 From the Normative to the Descriptive

The main formulations of contractualism in contemporary moral philosophy have now been introduced. Such theories are called normative because they are concerned with *what we ought to do* to behave morally. By opposition, scientific theories of moral cognition which will be discussed next are descriptive, as they are concerned with *what is:* understanding how moral cognition actually works. At this stage, one may therefore wonder: why take inspiration from normative ethical theories to construct descriptive accounts of morality? This section briefly outlines five key reasons to think this method can prove fruitful.

First, from an evolutionary perspective, human cognition has been shaped by evolutionary processes to approximate optimal solutions to specific recurrent problems faced in our ancestral environment (Cosmides and Tooby 2021). From this point of view, it is expected that a remarkably cooperative species like ours (Curry 2016; Tomasello and Vaish 2013) will have developed (over deep time) specific solutions to recurrent problems posed by cooperation, including the moral sense (Baumard 2016) or moral cognition more broadly. Such solutions will often obey principles of rationality or optimization put forward by normative accounts.

Second, basing descriptive accounts on philosophical theories has been a very successful strategy in moral psychology in recent decades. But despite this success, contractualism remains comparatively largely unexplored in empirical research on moral cognition. Most prominently, this general strategy has culminated in the influential dual‐process theory of moral judgment (Greene 2014; Greene et al. 2001). This theory proposes that our moral judgments are the result of two distinct and competing cognitive processes, which reflect the central distinction in moral philosophy between deontology and consequentialism. The first process is fast, automatic, intuitive, unconscious, largely driven by emotional reactions and leads to deontological moral judgments. The second process is slower, effortful, involves conscious and controlled deliberation, and makes judgments consistent with consequentialism. Deontology and consequentialism have received a lot of attention in empirical work on moral judgment and decision making (Bartels et al. 2015; Malle 2021). Virtue ethics, a third central family of theories in normative ethics which puts emphasis on virtues and moral character instead of rules and the consequences of actions (Hursthouse and Pettigrove 2023), has also inspired a substantial parallel literature in moral psychology (Uhlmann et al. 2015). But compared to deontology, consequentialism, and virtue ethics, contractualism had, until recently, received relatively little attention in empirical work.

Third, using normative models as a baseline for descriptive models has been a very successful approach in judgment and decision‐making research in general. Indeed, this strategy has made great strides, most prominently in the field of decision making under risk and uncertainty (Kahneman 2011; Tversky and Kahneman 1981). For example, prospect theory (Kahneman and Tversky 1979; Tversky and Kahneman 1992) builds on expected utility theory (Ramsey 1926/1931; Savage 1972; von Neumann and Morgenstern 1953) to develop a descriptive model of risky choice that can account for experimental evidence demonstrating the widespread empirical phenomenon of loss aversion. In fact, using normative theories either as first‐cut descriptive models, inspiration for descriptively richer psychological models, or as a benchmark against which to compare human judgments and decisions may be the standard approach in the field of judgment and decision making (Baron 2004; Bell et al. 1988). As moral judgments and decisions constitute some of the main outputs of moral cognition, this approach offers great promise for the study of morality, too.

Fourth, moral theories are often designed to be intuitively appealing and consistent with our ordinary moral judgments, at least in simple cases. And since everyday moral judgments and intuitions are precisely the object of study of descriptive approaches in moral psychology, it seems natural to use normative theories as a possible point of departure. One reason for this pertains to the methods employed by moral philosophers to construct and defend normative ethical theories, and in particular reflective equilibrium (Knight 2023). Reflective equilibrium involves working back and forth among one's particular moral intuitions and general moral principles and background theories—often with the help of carefully crafted thought experiments and counter‐examples—in order to achieve acceptable coherence among them (Daniels 1979; Rawls 1971). In fact, Baumard (2016, 4), referring to Scanlon (1998) points out that for some philosophers “if a moral theory is intuitive, that is a good reason to adopt it”. Turning this idea on its head, it seems sensible to think that the most prominent moral theories are likely to tap into laypeople's moral intuitions. And indeed, with a continued influence on moral theorizing tracing back to Protagoras, Epicurus (d'Agostino et al. 2011), and Plato (c. 375 bce/2007) and spanning centuries, the social‐contract tradition seems to appeal to some of our deepest moral intuitions. This provides us with a strong reason to investigate its descriptive relevance.

Fifth, among the main families of normative ethical theories, contractualism seems to enjoy a privileged position for forming the basis of a descriptive theory. Two simple observations support this idea. First, the contractualist tradition seems naturally equipped to understand why respecting promises, asking for consent, and abiding by written contracts are key components of our everyday lives (Levine, Chater, et al. 2024). Second, and perhaps most importantly, contractualism stands on three theoretical pillars that have stood the test of time and enjoyed widespread influence across the humanities, the natural sciences, and the social sciences: social‐contract theory, evolutionary theory, and game theory (Binmore 1994a, 1994b). These remarkably strong foundations arguably put contractualism in a unique position among its competitors in moral philosophy as a basis for theorizing in moral psychology.

## 3 Contractualist Moral Cognition at Three Levels of Analysis

**FIGURE 1:**
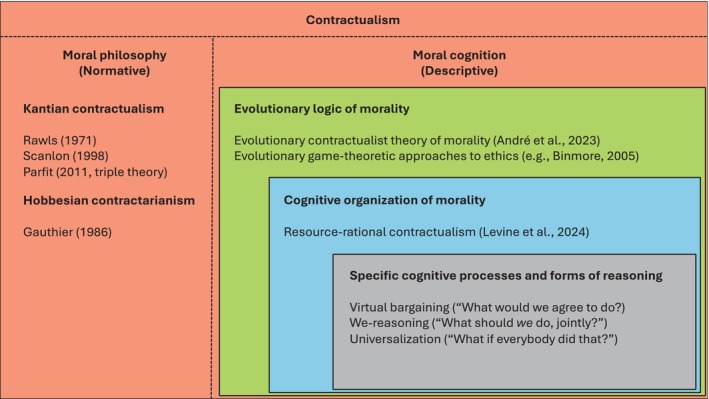
Visual summary of the paper. Taking inspiration from the contractualist tradition in moral philosophy is helpful to better understand morality at three interrelated levels of analysis: Its evolutionary logic, its cognitive organization, and its specific cognitive processes and forms of reasoning.

**FIGURE 2:**
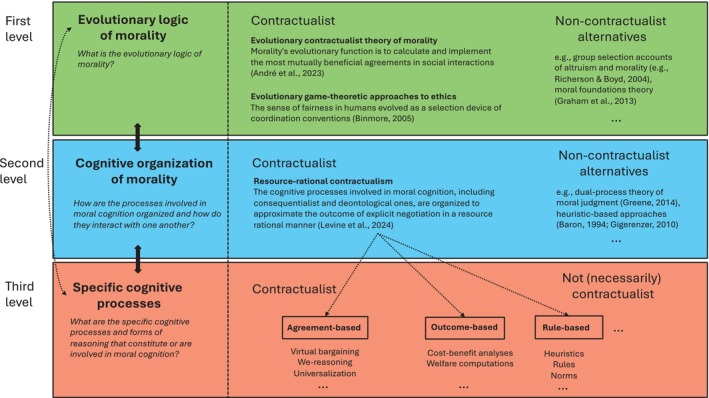
Contractualist approaches to moral cognition make contributions at three interrelated but distinct levels of analysis: understanding morality's evolutionary logic, mapping out how the various processes constituting moral cognition are organized and interact with one another, and identifying specific cognitive processes or forms of reasoning that may be involved in moral judgment and decision making.

This second section reviews how scientists have started to take inspiration from contractualism to construct descriptive accounts of morality. As we will see, adopting a contractualist outlook can be fruitful to shed light on moral cognition at three related but distinct levels of analysis: morality's evolutionary logic, its cognitive organization, and its specific cognitive processes, see Figures 1 and 2. All three levels interact with each other in a variety of ways, and it is not my intention to argue that their boundaries are always well defined. But distinguishing between them is helpful to map out and organize the variety of contributions that have been rendered possible by the contractualist metaphor.

### 3.1 Contractualist Evolutionary Logic of Morality

What is the evolutionary logic of morality? Various scholars have argued that morality's evolutionary function should be understood in contractualist terms. André et al. (2023) argue that morality's evolutionary logic is to calculate and implement maximally mutually beneficial agreements. For Binmore (2005), the sense of fairness in humans has evolved as a selection device of coordination conventions. In this section we primarily focus on these two accounts, which are both explicitly contractualist. To see why starting with this first level of analysis is important, consider that other evolutionary theories of morality have argued that it obeys a very different kind of logic, one that is not inherently contractualist. For example, group‐selection views of morality and altruism (Richerson and Boyd 2004; Sober and Wilson 1998) view groups as the relevant unit of (often cultural) selection processes for moral practices. They predict that morality will be a lot more altruistic and oriented towards the benefit of the group than would be expected under a contractualist outlook. Moral foundations theory (Graham et al. 2013) also posits an evolutionary basis of morality, but divides it into a plurality of logics corresponding to multiple recurrent adaptive problems faced by our ancestors. For this theory, the evolutionary logic of certain key aspects of morality is quite distant from a contractualist one (e.g., the adaptive challenge of sanctity/degradation is to avoid communicable diseases, for care/harm it is to protect and care for children (Graham et al. 2013)).

#### 3.1.1 Evolutionary Contractualist Theory of Morality

André et al. (2023)'s recent evolutionary contractualist theory of morality specifically purports to explain the existence, characteristics, and key features of moral cognition under a contractualist lens. In brief, the evolutionary contractualist theory of morality proposes that, because humans are under selective pressure to be recognized as good partners of cooperation by others, moral cognition evolved to constantly perform contractualist computations aimed at maximizing mutual benefit between cooperation partners.

This theory builds on earlier work by the same authors (Baumard 2016; Baumard et al. 2013) and involves three main steps. The first step is the observation that humans, as part of a cooperative species, are under selective pressure to be recognized as good partners of cooperation by others. In order to do so, they must make sure that others will not regret having cooperated with them. This means that, in social interactions, humans face a trade‐off between maximizing their immediate gains and minimizing their long‐term reputational costs. In a second step, the authors develop a formal approach to capture the relevant factors involved in solving this trade‐off—including opportunity costs, short‐term benefits, and long‐term reputational costs—and argue that the moral sense must navigate this trade‐off by maximizing the generalized Nash product of each social interaction. Third, from this analysis authors derive the theoretical proposition that, for each social interaction with opportunities for mutual benefit, the function of moral cognition is to compute the generalized Nash bargaining solution (i.e., the product of the difference between what the parties would get under the agreement of interest and what they would get in the absence of agreement that is, their disagreement payoff) of the underlying bargaining problem, and to ensure that each member of the interaction receives the payoff that corresponds to this solution (André et al. 2023)^3^.

The resulting view of morality is monist (as opposed to pluralist)—“morality obeys a single universal logic because it is an adaptive disposition whose function is to solve a set of specific problems that are only posed by reputation‐based cooperation and that are always posed by reputation‐based cooperation” (André et al. 2023, 9)—and grounded in the logic of mutual benefit. In addition, moral cognition is understood as a cross‐context contract calculator: “the moral sense has been shaped by natural selection to find every conceivable way for a set of individuals to generate mutual benefits” (André et al. 2023, 9). And this has the consequence that the moral sense is not limited to local and temporary contracts, but should also be activated for large‐scale contracts involving a large number of people (e.g., an entire community) and extended or indefinite periods of time. Finally, complementing this logic of mutual benefit is the phenomenology of duty. Moral cognition tags as morally wrong those behaviors that violate reciprocal contracts by not respecting the generalized Nash bargaining solution and, psychologically, the phenomenology that accompanies these computations is a (normative) sense of duty e.g., we feel the duty to behave morally even though we may, simultaneously, desire not to do so (e.g., we concurrently desire to cheat).

The authors contend that their theory can explain reciprocal obligations and moral intuitions in a variety of domains such as “distributive justice, ownership rules, the prohibition of violence, special obligations toward kin and ingroups, as well as moralistic punishment and obedience to authorities” (André et al. 2023, 1).

#### 3.1.2 Binmore's Natural Justice

Binmore uses the tools of game theory to develop a sophisticated evolutionary social‐contract theory of ethics (Binmore 1994a, 1994b, 2005). One of his primary goals is to provide an evolutionary account for the emergence of fairness. He takes social contracts to be the set of conventions that allow members of society to coordinate with one another, and views such conventions as the product of biological and cultural evolution (Binmore 2005). His argument involves the following claims. First, *stable* social conventions–those able to stand the test of time–are conventions that constitute equilibria of the repeated “game of life” played by societies. Second, among stable conventions, those that bring *efficient* outcomes will tend to predominate due to competition between groups and cultural transmission. Third, fairness norms are seen as the device by which we select among the variety of possible *efficient* and *stable* equilibria available to us.

Which fairness norms should we expect to prevail? For Binmore, when people make fairness judgments, “they are implicitly calculating the agreement that they would reach if they were to bargain under the assumption that their identities would be reassigned at random after the negotiation was over”, in other words the agreement that they would reach in Rawls' original position i.e., behind the veil of ignorance (Binmore 2005, 21). In order to figure out such agreements, people are assumed to engage in interpersonal comparisons of utilities. How they do so is culturally determined, even though some convergence is expected in the long run: “the standards of interpersonal comparison that are current in a society are determined by social or cultural evolution, with the result that any differences in the standards being operated tend to be ironed out in the long run” (Binmore 2005, 28). He calls these standards “social indices”, which act as inputs to specific bargaining problems.

According to Binmore, agreements predicted by the egalitarian bargaining solution (which in his setting ends up coinciding with the Nash bargaining solution) should prevail in the absence of an external agency to enforce hypothetical contracts. This is the situation that corresponds to the conditions under which our sense of fairness is most likely to have evolved. This solution favors individuals with higher social indices and higher “disagreement payoffs” (which reflect the *status quo* or current social contract in place). In his theory, social indices are taken to increase with one's ability and “need” (i.e., here understood as leading to being risk‐seeking and “tougher” in the negotiation) and decrease with status and effort, whereas disagreement payoffs increase with status. These inputs suggest an important role played by power dynamics in the output of fairness judgments: “the social indices that characterize a fairness norm will reflect the historical accidents frozen into the power structure of the social contract currently being operated” (Binmore 2005, 180).

#### 3.1.3 Other Evolutionary Game‐Theoretic Approaches to Ethics and Cooperation

In the preceding paragraphs we have focused on two evolutionary accounts of morality and fairness that are explicitly contractualist. Importantly, there is of course a long and rich history of research at the intersection of evolutionary game theory, cooperation, and ethics, which is broadly compatible and in spirit very close to the contractualist outlook. As opposed to focusing on bargaining solutions (as above), these analyses usually centre around the concept of (Nash) equilibrium. While these accounts cannot be reviewed in detail here, some key approaches are briefly introduced below.

With regards to the evolution of cooperative behavior broadly understood, seminal work that has applied the tools of game theory includes analyses of the evolution of reciprocal altruism (Trivers 1971), cooperation (Axelrod and Hamilton 1981; Nowak 2006), animal conflict (Smith and Price 1973), and punishment (Boyd and Richerson 1992). Regarding morality specifically, the theory of morality as cooperation proposes that we can derive the domains of morality (i.e., the key problems of cooperation that are recurrent in social life), and the cultural and biological mechanisms that constitute it, from game theory (Curry 2016; Curry et al. 2022, 2019). For a recent application of game‐theoretic tools to morality, see also Hoffman et al. (2016). Finally, a parallel line of research that often characterizes the evolutionary logic of ethics in contractualist (or more precisely contractarian) terms is found in contemporary philosophy of science. These approaches, which build on influential accounts of convention developed by Hume (1739/2000) and Lewis (1969), include analyses of the evolution of social norms (Bicchieri 2005), social conventions (Vanderschraaf 2018), social contracts and cooperation (Skyrms 2014), and unfairness (O'Connor 2019). Some of these involve blends of descriptive and normative claims, focusing on explaining the emergence and stability of certain moral norms and practices, and sometimes on justifying and defending them. However, their primary focus is usually not to describe moral cognition or the functioning of psychological processes, making them less central for our immediate purposes. For an introduction to this literature, interested readers can consult Hankins and Vanderschraaf (2021).

### 3.2 Contractualist Cognitive Organization of Morality

If the evolutionary logic of morality is contractualist, how are the processes involved in moral cognition organized and how do they interact with one another? Resource‐rational contractualism (Levine, Chater, et al. 2024) posits that the heuristics and abstract representations that form the moral mind–such as outcome‐, rule‐, and agreement‐based mechanisms–are organized to produce cognitively efficient or resource‐rational approximations of a contractualist ideal. This second level of analysis is key because influential alternative theories have previously proposed a very different kind of organization for moral cognition. For example, the dual‐process theory of moral judgment posits two main systems that are in conflict with one another: quick and emotional processes tend to produce characteristically deontological judgments whereas slower and more deliberative processes tend to produce consequentialist judgments (Greene 2014). Moreover, other scholars have proposed that the heuristics that underlie moral decision making are overgeneralizations of consequentialist solutions (Baron 1994), or are directed at fostering the social coherence or coordination of groups (Gigerenzer 2010), implying a cognitive organization of morality that is not inherently contractualist.

#### 3.2.1 Resource‐Rational Contractualism

Resource‐rational contractualism (Levine, Chater, et al. 2024) provides a framework to understand the architecture and organization of the cognitive processes and mechanisms involved in moral cognition. The authors' main claim is that moral cognition can be seen as approximating the outcome of explicit negotiation between relevant parties in a cognitively efficient way under limited resources. Their argument involves several steps.

To begin with, the authors draw on the observation that there is widespread agreement among various disciplines (including psychology, anthropology, economics, and biology) that morality has evolved to solve the problem of cooperation between agents with conflicting interests. This seems to imply that, ideally, we would solve the problem of “interdependent rational choice” via explicit bargaining and negotiation. But of course, in practice, constraints on individual cognition, computational resources, and social coordination prevent us from engaging in literal negotiation all the time. Thus, a psychologically plausible account of contractualism should go beyond the modeling of ideal bargaining processes between perfectly rational and/or impartial agents to incorporate these constraints. To do so, authors combine contractualism with resource rationality, a cognitive modeling paradigm for reverse‐engineering psychological mechanisms and representations by assuming that cognition makes “optimal use of limited computational resources” (Lieder and Griffiths 2020, 1).

Under the resulting view, people are seen as having two main ways to efficiently approximate the ideal of negotiation. First, they can use “model‐based” methods (e.g., virtual bargaining, universalization, weighted cost–benefit calculations)—for example, by mentally simulating a negotiation process or imagining a discussion between the contracting parties and implementing its outcome (Levine, Kleiman‐Weiner, et al. 2024). While less socially demanding than actual bargaining, these still require substantial computational resources. Alternatively, people can use “model‐free” methods, for example, by acting based on precedent and/or generalizing past agreements. Using simple heuristics or rules (e.g., certain actions are forbidden) or following established norms (e.g., dividing resources equally) is cognitively very efficient but typically leads to less precise approximations of the solution that would arise through actual bargaining. Importantly, whether they do so via (precise but cognitively costly) model‐based or (imprecise but cognitively efficient) model‐free methods, people can bargain at different levels of abstraction. In other words, the *terms* of the agreement themselves can be more or less abstract, covering either what to do in specific instances (i.e., bargaining about precise actions to perform here and now), welfare standards (i.e., bargaining about weights to attach to each party's welfare in a range of contexts), or action standards (i.e., bargaining about general rules, norms and principles to adopt) (Levine, Chater, et al. 2024).

Resource‐rational contractualism provides two important theoretical benefits. First, it offers a way to integrate two well‐studied but conflicting aspects of our moral minds—deontological thinking, which assigns constraints to certain actions, and consequentialist thinking, which performs weighted utility calculations to determine the right course of action—into a single contractualist logic, by showing that these constitute two efficient ways to approximate the outcome of ad hoc negotiation. The resulting “triple theory” can thus elegantly unify deontological, consequentialist, and contractualist concerns into a single framework^4^. Second, it provides an explanation for moral flexibility. Moral flexibility refers to our capacity to revise and update our moral standards. This happens when the environment changes and previous norms can no longer be applied, or when we come up with exceptions to existing rules and principles. Resource‐rational contractualism proposes that we update our moral norms when (re‐)negotiation would provide a better way to achieve mutual benefit. Thus, both the establishment and revision of moral standards occur via (virtual or actual) bargaining processes aimed at efficiently maximizing mutual benefit between parties with conflicting interests (Levine, Chater, et al. 2024).

### 3.3 Specific Contractualist Cognitive Processes

That moral cognition is organized around contractualist principles does not entail that the cognitive processes involved in moral judgment and decision making must themselves be agreement‐based. After all, as Levine, Chater, et al. (2024) note, rule‐ and consequence‐based mechanisms sometimes can, in principle, lead to contractualist outcomes. What are the specific cognitive processes that play a role in producing moral judgments and decisions? In this section we will see that taking inspiration from contractualism can also be fruitful to identify specific cognitive processes and forms of reasoning. Three agreement‐based cognitive processes that have attracted empirical attention recently are highlighted: virtual bargaining, we‐reasoning/joint commitments, and universalization. This third level of analysis is also fundamental, as many traditionally studied mechanisms in moral psychology–such as cost–benefit analyses (e.g., “is it ok to kill one in order to save five?”) or rule‐based thinking (e.g., “what he did is wrong because it violates the law”)–are not explicitly based on agreement or negotiation.

#### 3.3.1 Virtual Bargaining

According to virtual bargaining theory (Chater et al. 2016, 2022; Misyak and Chater 2014; Misyak et al. 2014) humans often interact and coordinate with one another “on the basis of what they would agree to do if they were explicitly to bargain” (Misyak et al. 2014, 512).

This can be done by mentally simulating (parts or the outcome) of a negotiation process or discussion between relevant parties, a form of reasoning also called virtual bargaining. Thus, before making decisions in the context of a social interaction, people can ask themselves “what would we agree to do?” or “would the other person agree if I do X?”. Alternatively, they can also consciously or unconsciously figure out the outcome of such a bargaining procedure and directly implement it. In other words, they can infer the joint plan to be followed and play their role in it (Chater et al. 2022).

Because virtual bargaining consists in figuring out what rational agents would agree to do if they were to negotiate, this form of reasoning is fundamentally contractualist. While the ensuing simulation can sometimes be explicit—with decision makers imagining what other parties would say—often, virtual bargaining reasoning does not require conscious or verbalized thinking. Instead, people behave *as if* they had been able to negotiate (Le Pargneux et al. 2024).

At this stage it is thus useful to distinguish between two usages of the term virtual bargaining. “Fully‐fledged” virtual bargaining involves explicitly imagining a bargaining *procedure* (e.g., a series of offers and counter‐offers; a conversation between two parties). This is likely to be quite rare and cognitively costly (e.g., limited to high‐stakes negotiations). By contrast, “minimal” virtual bargaining may simply involve simulating the *outcome* of a negotiation process, or a mutually beneficial arrangement (e.g., a 50–50 split), and implementing it. This will often be fairly immediate, involving little conscious thinking, if any (e.g., as when two people coordinate to jointly move a couch).

Virtual bargaining theory has received some empirical support and has been applied to multiple domains, including the study of tacit coordination (Misyak and Chater 2022; Misyak et al. 2016), reinterpretation of key phenomena in game‐theoretic settings (Melkonyan et al. 2018, 2022; Misyak and Chater 2014), and explanation of central features of language and non‐verbal communication (Christiansen and Chater 2022). It has also been formalized in game‐theoretic terms under the concept of virtual bargaining equilibrium (Melkonyan et al. 2018, 2022; Misyak and Chater 2014).

#### 3.3.2 Relevant Empirical Evidence

Most importantly for our purposes, the role of virtual bargaining in moral judgment and decision making has been explored in a series of recent empirical papers.

First, in several experiments, Levine, Kleiman‐Weiner, et al. (2024) presented participants with a moral dilemma in which a stranger offers a character a sum of money (e.g., $1 million) for altering someone's property (e.g., re‐paint their neighbor's house in blue) without their permission. A substantial proportion of participants viewed it as morally acceptable to accept the stranger's offer and thus to break the simple rule “only the owner of a property has the right to use, modify or destroy it” (Levine, Chater, et al. 2024, 4). Crucially, these moral judgments depended on whether the amount offered was sufficiently large to compensate the affected party (e.g., the neighbor) for the property violation with a side‐payment. Using data on expected compensation and projected side‐payments, the authors showed that an agreement‐based model of virtual bargaining was able to explain key aspects of people's judgments in variants of this task, but that more traditional alternative models based on utilities or rules could not. Moreover, people were more (less) likely to break the rule when the neighbor was described as reasonable (unreasonable) and likely (unlikely) to agree to having his house repainted. People also judged it morally best to compensate the directly affected party, as opposed to donating the money to charity or to another victim of a similar property violation, inconsistent with alternative explanations based on social preferences. Overall, these findings are consistent with some participants engaging in virtual bargaining processes in this dilemma, reasoning about whether it is acceptable to break the rule if doing so would be mutually beneficial overall.

Second, building on the same paradigm, Trujillo et al. (2024) developed a probabilistic resource‐rational model of virtual bargaining for this task. Their model assumes that people will engage in cognitively effortful virtual bargaining reasoning when the stakes are higher, and revert to cognitively cheap rule‐based thinking when the stakes are lower, consistent with resource‐rational principles. The authors systematically manipulated stakes and fitted their computational model to the data. In model comparisons, they provide evidence that the resource‐rational component of their model captures important aspects of the data, with about a third of participants flexibly switching between rule‐based thinking and virtual bargaining, a third of participants using virtual bargaining only, and another third of participants using rule‐based thinking only.

Third, Le Pargneux et al. (2024) had people play an incentivized lottery game which shares an analogous structure to Levine, Kleiman‐Weiner, et al. (2024)'s dilemma. In this game, players could destroy another player's ticket without their permission in order for extra lottery tickets to be distributed. The majority of participants viewed destroying the other player's ticket and splitting any extra lottery tickets with them as the morally best move among several alternatives, which included a characteristically deontological (refusing to destroy the ticket), consequentialist (donating extra tickets of higher value to a third player), and egoist option (keeping all extra tickets). Results were consistent with virtual bargaining influencing both incentivized decisions and moral judgments in this task, with a substantial proportion of players making choices corresponding to what the players would be likely to agree to do if they could talk. Le Pargneux et al. (2024) then developed a separate economic game in which two players could obtain asymmetric monetary rewards if they successfully coordinated about who should “press a button” without talking. In this task, participants could tacitly agree to use the outcome of a colored dice roll as a coordination device to determine what to do. By varying the dice roll's outcome, the experimenters could thus manipulate the presence or absence of a potential tacit agreement or virtual bargain between the players. Incentivized decisions and third‐party moral judgments were sensitive to whether such a tacit agreement was available or not, the same action being perceived as less (more) morally appropriate when it violated (respected) the virtual bargain, compared to when no virtual bargain was available.

Fourth, Le Pargneux and Cushman (2025) developed 12 scenarios in which two protagonists can mutually benefit if either of them performs an unpleasant action (e.g., two colleagues sharing a cab and deciding who should be dropped off first, two partners deciding who should travel to the client's office). The characters only differed in terms of their bargaining power, which was manipulated either via the stakes that each of them had in the potential agreement or the alternatives available to them in the absence of agreement. Participants were asked about the moral appropriateness of asking the other party to perform the unpleasant action, or of explicitly refusing to do it. In five experiments and using five measures of moral judgment, the authors found that people's moral judgments systematically advantaged the party with higher bargaining power, consistent with participants implicitly tracking bargaining power asymmetries in social interactions, shaping their evaluations of what is morally appropriate or not.

Overall, this series of papers provides empirical evidence that moral judgments and decisions in various contexts can be influenced by “minimal” virtual bargaining processes (e.g., mental simulations of social arrangements that would bring about mutual benefit). Importantly, however, it is unclear at this stage whether and to what extent more “fully‐fledged” virtual bargaining processes (e.g., explicitly *imagining* a bargaining procedure) are involved in such tasks.

#### 3.3.3 We‐Reasoning and Joint Commitments

Virtual bargaining is one among several forms of “we‐reasoning” (Bacharach 2006; Chater et al. 2022). We‐reasoning can be described as follows: “People sometimes think in terms of ‘we’ referring to a group they belong to. When making decisions, they frame the decision problem as: ‘What should we do?’ instead of ‘What should I do?’” (Hakli et al. 2010, 291). This “we” can refer to different types of plural agents: a couple, a family, a team, a group, an organization, a nation etc.

Conceptually, the idea of we‐reasoning has been discussed in a variety of disciplines–including the philosophy of joint action, developmental and comparative psychology, and game theory–under different forms. Here, the term “we‐reasoning” is used in a broad sense to encompass various approaches to joint or collective reasoning including we‐mode reasoning, joint actions, joint commitments, shared intentionality, team reasoning, and virtual bargaining. These concepts are introduced below, and the relevant empirical evidence related to morality is presented next. Importantly, we‐reasoning is a general‐purpose form of reasoning facilitating coordination in a range of contexts that may be involved in moral cognition but is not specific or restricted to it.

#### 3.3.4 In the Philosophy of Joint Action

In philosophy, discussions have focused on the concepts of joint actions and of reasoning in the “we‐mode”.

Several scholars have argued that *collective* or *joint* actions are not reducible to the summation of individual intentional actions (Bratman 1992; Gilbert 2006; Searle 1990). Examples of collective or joint actions are numerous: seeing someone pushing a car in the street and spontaneously pushing with him, a NFL team executing a pass play (Searle 1990), marching or walking together, dancing together, making love together, making music together, or painting a house together (Gilbert 2006). Of direct importance–as it relates to some of the empirical evidence presented below–is the concept of joint commitment (Gilbert 2013). What is a joint commitment? According to Gilbert, when one decides to do something, one is committed to it and “reason requires one to act in accordance with that commitment, all else being equal.” Personal commitments are those that are brought into existence by one person only. By analogy, a joint commitment, which does not have to be explicit, is defined as “the commitment of two or more people” in which “the participants must be jointly committed to espousing a goal as a body” (Gilbert 2013, 31, 32).

Tuomela (2007) makes a distinction between reasoning in the I‐mode and in the we‐mode, where the I‐mode refers to thinking and acting as a private person, and the we‐mode refers to thinking and acting as a proper member of a group (Hakli et al. 2010). Specifically, we‐mode reasoning is concerned with two types of questions: “What should our group do?” and “What should I do as a group member as my part of our group's action?” (Hakli et al. 2010, 293).

#### 3.3.5 In Developmental and Comparative Psychology

In developmental and comparative psychology, “shared intentionality” refers to the specifically human ability to participate in collaborative activities with shared goals and intentions (Tomasello et al. 2005) and has been defined as “collaborative interactions in which participants have a shared goal (shared commitment) and coordinated action roles for pursuing that shared goal” (Tomasello et al. 2005, 680). The available evidence suggests that children are uniquely skilled for shared intentionality, but that great apes are unable to participate in activities involving joint intentions and attention (Tomasello et al. 2005). A key feature of shared intentionality is to share psychological states with one another: joint attention is an intersubjective activity which involves common psychological ground and occurs when parties attend to the same thing, and know that they are attending to the same thing (Tomasello and Carpenter 2007).

#### 3.3.6 In Game Theory

In game theory, Schelling (1960) has proposed that, in games with more than one Nash equilibrium, one equilibrium—the focal point—is often more salient than the others, stands out, and is recognized as such by the players (Mehta et al. 1994). Experimental evidence confirms that, without the possibility to communicate, people are remarkably good at coordinating using focal points in a variety of games (Bardsley et al. 2010; Mehta et al. 1994).

Building on the idea of focal points, team reasoning is a theory of decision making in game theory which proposes that, instead of engaging in individualistic best‐response reasoning, individuals sometimes reason from the perspective of a team that is, as a group of individuals with distinct roles to achieve the best outcome for the group (Karpus and Gold 2016). In other words, instead of asking “What do I want?” or “What should I do to achieve this?” players decide as if by asking “What do we want?”, “What should we do?” or “What should I do to play my part in achieving this?” (Colman and Gold 2018, 1174). This distinct form of strategic reasoning involves a shift from individual agency to group agency, though the decision is still taken by the players as individuals (Colman and Gold 2018). Several theories of team reasoning have been proposed (Bacharach 1999, 2006; Sugden 2003) and received empirical support (Bardsley et al. 2010; Colman et al. 2014, 2008; Mehta et al. 1994).

#### 3.3.7 Relevant Empirical Evidence

In many circumstances, “what *we* should do” or “what *we* are committed to do” will be indistinguishable from “what we would *agree* to do” or “what we have *agreed* to do”. Because of these deep links to tacit or implicit agreements, we‐reasoning processes seem to have a clear contractualist flavor. Does we‐reasoning play a role in moral cognition? The most relevant published empirical evidence on this question comes from the study of joint commitments in the developmental psychology literature (see also Tomasello (2020)), which is the main focus of this subsection.

Do pre‐school children understand joint commitments? A first line of inquiry provides evidence that young children have the capacity to understand when they are committed to a joint activity, and that this has specific implications for how they think they and others should behave. In one study, Gräfenhain et al. (2009) had 2‐and 3‐year‐olds play a game with an adult. During the game, the adult unexpectedly stopped to play with the child. Three‐year‐olds, but not 2‐year‐olds, tried to re‐engage the adult back into the game more often when a joint commitment had previously been established via an explicit invitation to play the game together (vs. a control condition). In another study, 3‐and 4‐year‐olds were enticed away from an activity with an adult. In both age groups, children were more likely to acknowledge their leaving (e.g., by looking at the adult or handing them a tool required to play the game) following a joint commitment, compared to a control condition. This suggests that by 3 years of age, children have an understanding of when they are committed to a joint activity.

What do joint commitments imply? Hamann et al. (2012) had 2.5 and 3.5‐year‐old play a game in pairs. In the game, one child received an early reward and had to continue to collaborate in order for the other child to receive a reward. Older but not younger children were more likely to continue collaborating (relative to a control condition with a non‐collaborative version of the task) suggesting that they felt a sense of normative obligation or commitment when playing this collaborative task. Moreover, Gräfenhain et al. (2013) had 3‐year‐olds play a puzzle with a puppet partner. They either had both partners agree to play the puzzle together (collaborative condition) or both partners were encouraged to play a puzzle on their own in parallel (control). Children were more likely to wait for their partner, spontaneously help them, or take over their role in the collaborative condition than in the control condition. There was no effect of the manipulation on tattling behavior or on tendencies to distribute rewards equally. The authors argue that by 3 years of age, joint commitments create a “we”‐intentionality in children during joint activities.

Do joint commitments result in a felt sense of obligation or normativity? Expanding on this line of research, Kachel et al. (2018) had pairs of 3‐year‐old children make a joint commitment by agreeing to play a specific game together. In all conditions, one child was trained to play the game properly. In three separate conditions, the other child was trained to play the game differently such that, when they both played together, the game appeared to have been interrupted for one of three reasons: either the second child had intentionally defected, was ignorant about how to play, or the apparatus accidentally broke. Children were more likely to make normative protests (e.g., “that's wrong”), were more emotionally aroused (signs of irritation, frustration, anger), and were more likely to blame their partner (tattling) in the intentional defection condition than in the other conditions. This is consistent with intentional defections from joint commitments eliciting “moral” reactions from an early age. Second, Kachel and Tomasello (2019) had pairs of 3‐and 5‐year‐olds play a collaborative game, each time offering one child a bribe to stop playing the game. In three conditions, children committed to playing the game together either explicitly or implicitly, or simply played in parallel. 3‐year‐olds were more likely to accept a bribe as the level of commitment decreased. 5‐year‐olds were less likely to accept bribes in the implicit and explicit commitment conditions, compared to the no commitment condition. This suggests that implicit joint commitments can create a sense of obligation even in preschool children. Third, in a subsequent study, Kachel et al. (2019) had 3‐and 5‐year‐olds form a joint commitment with a puppet to play a game together. In three conditions, the puppet either left the game abruptly, after notifying the child, or after asking for the child's permission and waiting for his agreement to stop playing. Children protested the least and were least likely to say that the puppet deserved scolding when they had agreed to the puppet leaving the game than in the other conditions. This suggests that from an early age, children can understand that the obligations of a joint commitment may be dissolved by mutual agreement. Finally, Winter and Tomasello (2024) had 3‐and 5‐year‐olds play a game with a peer using recorded videos where the peer states their preferred way of playing the game out of two options. In each case, the subject and the peer's preferred way of playing the game differed. In one condition, the experimenter had the children explicitly agree to play according to their peer's preference. In a control condition, no such agreement was elicited. 5‐year‐olds, but not 3‐year‐olds, were more likely to abide by this agreement that went against their initial preference, suggesting that they felt a sense of obligation to play as agreed.

Can a “sense of we” be verbally induced? A parallel line of investigation tackles this question. Vasil and Tomasello (2022) attempt to verbally induce a “normative sense of we” in 3 and 4‐year‐old children. Children drew with a puppet which either repeatedly framed the activity using the pronoun “we” or “you”. While the results are somewhat ambiguous, they suggest that children felt a greater sense of commitment to their partner in the “we”‐framing condition than in the “you”‐framing condition, by being more reluctant to leave for a more fun game (4‐year‐olds) or more likely to announce their leaving (3‐year‐olds). No difference in helping or sharing behavior was observed. In a subsequent study (Vasil et al. 2024), 2 and 3‐year‐olds played a boring game with either one or three puppets and had the opportunity to abandon their partners for a more fun game. The puppet(s) either repeatedly framed the game using the pronoun “we” or “you”. The authors predicted that children would remain longer with their partner under we‐framing. While they interpret their results as reliably influencing 2‐year‐olds' sense of commitment, the findings are mixed and the evidence seems inconclusive at this stage.

Overall, findings from the above research program provide empirical evidence that points towards a link between joint commitments and a sense of obligation or normativity in pre‐school children. While the evidence is stronger for an influence of *explicit* joint commitments and agreements on children's “normative” or “moral” reactions, there is also preliminary evidence suggesting that *implicit* joint commitments, and perhaps “we‐framing”, may play a role too. This would be consistent with we‐reasoning being involved in moral cognition from an early age. Importantly, caution is still warranted at this stage given the small sample sizes (inherent to research in developmental psychology) and sometimes mixed results in previously described studies. In addition, future research should test whether implicit joint commitments can influence moral judgments in adults.

#### 3.3.8 Universalization

Beyond virtual bargaining and we‐reasoning, a third form of contractualist reasoning is universalization (Levine, Chater, et al. 2024; Levine et al. 2020). When deciding, we often ask ourselves “What if everybody did that?”. We may do so for example to figure out whether an action is permissible (e.g., “is it permissible not to vote at the next election?”) or to decide whether a novel rule should be adopted (e.g., “it should be forbidden to litter in this area”) (Levine et al. 2020). This decision procedure, which is at the core of Kant's moral philosophy (Kant 1739/2000), has contractualist accents (Levine, Chater, et al. 2024; Parfit 2011; Rawls 1971; Scanlon 1998) as it is akin to figuring out what all of us—e.g., as members of a community, society, or humanity as a whole—could agree to.

#### 3.3.9 Relevant Empirical Evidence

Recent experimental work (Levine et al. 2020) provides evidence that adults and children make moral judgments consistent with the logic of universalization in “threshold problems”. In such contexts, if only a few people perform a certain act (for example, hunt or harvest with a novel technology), the outcome is mutually advantageous, but if the threshold is reached, resources are depleted for everyone. Levine et al. (2020) presented participants with variants of a threshold problem in which a protagonist can start fishing in a lake using a new hook. If a certain number of people start using this new hook, the fish population will collapse. In a careful set of experiments, the authors show that people's moral judgments are sensitive to the number of parties interested in using the new hook, the presence or absence of a threshold, and how everyone using the lake would be affected, as uniquely predicted by universalization. Importantly, alternative models based on rules, outcomes, or norms cannot account for all three effects. The authors also fit a computational model of universalization to participants' data, showing that it reproduces key qualitative patterns in observed moral judgments. Finally, using an analogous scenario, the authors find that acceptability judgments made by children between 4 and 11 years old are also sensitive to the number of interested parties, consistent with universalization and with adults' judgments.

Furthermore, Awad et al. (2024) also find evidence for a role played by universalization mechanisms in rule‐breaking judgments in a different setting: waiting in line. They assigned participants to one of three conditions where participants were either asked to imagine standing in line at a deli, a single‐occupancy bathroom, or an airport. The experimenters manipulated various parameters, such as how beneficial it would be for the protagonist to cut the line, or the time delay for other people if they did. Participants made a judgment about whether it is acceptable to cut the line in this context and were asked a variety of questions about the action's consequences, including the overall utility consequences if this type of line‐cutting was always allowed (i.e., universalized). Authors found that the universalization question had the strongest negative correlation with acceptability, suggesting that this consideration played an important role in influencing moral judgments. They interpreted this and additional results as providing evidence that this contractualist form of reasoning is at play in figuring out when to break rules about waiting in line.

#### 3.3.10 Other Agreement‐Based Cognitive Processes

While virtual bargaining, we‐reasoning, and universalization have attracted very recent empirical attention, they are not the only agreement‐like cognitive processes that have been posited to influence moral judgment. Here I briefly mention two other candidates, the cheater detection system (Cosmides and Tooby 2015) and the recalibrational theory of anger (Sell et al. 2017). For introduction to the empirical evidence in their support, see respectively Cosmides and Tooby (2015) and Sell and Sznycer (2024).

An early and influential appeal to the notion of social contract in the analysis of forms of moral reasoning is Cosmides and Tooby's “social contract theory” (Cosmides 1989; Cosmides and Tooby 2008). In brief, this theory is developed to analyze cheater detection in situations of mutually beneficial social exchange. It is argued that to detect cheaters in the context of two‐party reciprocation, the human mind must be able to represent costs and benefits and to be equipped with a capacity for conditional reasoning of the form “If you accept benefit B from me, then you must satisfy my requirement R” (Cosmides et al. 2018, 199). An individual who takes a benefit without satisfying the relevant requirement is detected as a cheater and evaluated negatively. This form of conditional reasoning which performs a specialized moral function, is inherently agreement‐based.

In addition, bargaining and negotiation processes have also been posited to play a crucial role in the functioning of anger, a key moral emotion. According to the recalibrational theory of anger, anger constitutes a cognitive system evolved to leverage one's bargaining position in order to obtain better treatment from others (Sell et al. 2017, 2009; Sell 2011). When making decisions that impact ourselves and others, we need to trade off our own welfare versus the welfare of others. The concept of welfare trade‐off ratio captures the relative weight that we assign to someone else's welfare compared to our own. The higher the welfare trade‐off ratio that another person assigns to me, the better the treatment I receive from them e.g., the lower the cost they would be willing to inflict on me to receive a certain benefit, all other things equal. The recalibrational theory argues that we use anger to negotiate better welfare trade‐off ratios from others we interact with when conflicts of interests arise (Sell et al. 2017). While not of explicit contractualist inspiration, this theory provides a contractualist analysis of a central moral emotion.

Overall, we have seen in this section that adopting a contractualist lens can be fruitful to identify specific forms of reasoning that may be involved in moral cognition, such as virtual bargaining, we‐reasoning and joint commitments, and universalization, or shed light on processes involved in specialized moral functions such as cheater detection or the functioning of moral emotions like anger.

## 4 Conclusion

Contractualism refers to a family of theories in moral philosophy for which morality consists in what rational agents in search of mutual benefit would agree to do. Its main contemporary proponents include Rawls, Gauthier, Scanlon, and Parfit. In the study of moral cognition, adopting a contractualist framework has recently proven fruitful at three interrelated levels of analysis. First, André and colleagues have defended that morality's ultimate evolutionary logic lies in helping us appear as good cooperative investments by maximizing mutual benefit in our interactions. In a related vein, Binmore has proposed to understand the evolution of the sense of fairness in humans in contractualist (Rawlsian) terms. Second, Levine and colleagues have argued that we must understand the cognitive organization of the heuristics and abstract representations involved in moral judgment as designed to efficiently approximate the outcome of actual negotiation under resource constraints. Third, the contractualist lens also identifies candidates for specific cognitive processes and forms of reasoning at play in moral judgment and decision making. Empirical support for an influence on moral cognition of three of such processes—virtual bargaining, we‐reasoning/joint commitments, and universalization—has been found in very recent work. Beyond the traditional distinction between rules and consequences, contractualist approaches to moral cognition can offer a third way for a cognitive science of morality, one based on agreement.

This review is organized according to three levels of analysis–evolutionary function, cognitive organization, and specific cognitive processes themselves–in order to highlight the various types of contributions rendered possible by the contractualist metaphor. Evidently, all three levels interact with each other in various ways and the boundaries between them are not always clear‐cut. And crucially, several theories discussed in this review make contributions at more than one, or at all levels (André et al. 2023; Binmore 2005; Levine, Chater, et al. 2024). Still, the proposed distinction seems helpful to map out recent developments in contractualist approaches to morality. To see why, consider that a unified theory of moral cognition could in principle posit a contractualist evolutionary logic without positing the existence of any agreement‐based forms of reasoning. Or that it could posit an important role of agreement‐based processes in a dual‐process organization that is better described as divided along deontological and consequentialist logics. Or even that moral psychology is indeed organized to approximate explicit negotiation but not as the result of any evolutionary process–rather being shaped entirely by learning, communication, and social mechanisms only.

Despite the recent theoretical and empirical progress highlighted in this review, investigations of contractualist moral cognition are still in their infancy, and more research is needed. Promising avenues for future research include experimental tests of specific predictions made by resource‐rational contractualism (Levine, Chater, et al. 2024), game‐theoretic simulations of the biological and cultural evolution of specific features of contractualist morality, development of computational models of contractualist forms of reasoning, big data analyses of contractualist morality based on historical and anthropological records, or empirical investigations of the moral psychology of large‐scale (group‐ or society‐wide) social contracts (e.g., Korn et al. (2020)). A contractualist approach also raises many interesting questions for future work, a few of which are highlighted below. Are there other types of agreement‐based forms of reasoning involved in moral cognition than those identified in this review? What are the specific implications of explicit agreements for moral cognition, over and beyond implicit ones? Why are some explicit agreements (e.g., resulting from coercion or threats) sometimes seen as unjust, exploitative, or immoral (Levine, Chater, et al. 2024)? How well does the contractualist framework capture moral judgments outside of bargaining or resource division contexts? How culturally variable are contractualist forms of reasoning? What is the relationship between contractualist morality and judgments of moral character (Everett et al. 2016)? What is the relationship between contractualist forms of reasoning and the tension between impartiality (Huang et al. 2019) and partiality (Le Pargneux and Cushman 2025) in moral cognition? How can we implement contractualist reasoning or moral principles in artificial agents? These questions, and many others, constitute exciting immediate challenges for the burgeoning literature on contractualist moral cognition.

## Author Contributions

**Arthur Le Pargneux:** conceptualization (lead), data curation (lead), formal analysis (lead), funding acquisition (lead), investigation (lead), methodology (lead), project administration (lead), resources (lead), software (lead), supervision (lead), validation (lead), visualization (lead), writing – original draft (lead), writing – review and editing (lead).

## Conflicts of Interest

The author declares no conflicts of interest.

## Related WIREs Articles

Emotion and moral judgment

Punishment

Moral psychology (ethics)

An integrative cognitive neuroscience theory of social reasoning and moral judgment

## Data Availability

Data sharing is not applicable to this article as no new data were created or analyzed in this study.

## Endnotes

^1^
Scanlon carefully distinguishes between *rationality* (“what most conduces to the fulfillment of an agent's aims”) and *reasonableness* (“A claim about what it is reasonable for a person to do presupposes a certain body of information and a certain range of reasons which are taken to be relevant” (Scanlon 1998, 191, 192)). This fine‐grained distinction within his theory is unlikely to be of primary importance from a descriptive point of view.

^2^
Note that Kant is a central figure for both deontology and contractualism. And, in the same way that demarcating consequentialism and deontology is sometimes delicate (e.g., rule consequentialism) (Le Pargneux et al. 2024), the boundaries between deontology and certain contractualist theories from the Kantian tradition, such as Scanlon's, are not always clear‐cut (Everett et al. 2016).

^3^
This form of contractualism is consequentialist in the sense that actions are evaluated in terms of their consequences only. As opposed to utilitarian approaches focusing on aggregate welfare, this contractualism aims to maximize mutual benefit (e.g., the Nash product) instead.

^4^
An alternative (perhaps complementary) way to reconcile deontological and consequentialist tendencies is to view rules as predictable commitments used to facilitate coordination with others, including perfect strangers. Seen as large‐scale coordination devices, rules lead to the maximization of mutual benefit once indirect (as opposed to immediate, in‐the‐moment) consequences are taken into account (André et al. 2023).
